# Surgery

**Published:** 1906-12

**Authors:** Harold F. Mole


### SURGERY.

Cases of **stone in the ureter** are now fairly frequently reported, and although the methods of diagnosis have been considerably improved upon of late, still great difficulty often arises first of all in stating that there is probably a calculus present, and secondly in locating its exact situation in the ureter. The X-rays have often been of considerable value, but a negative finding is useless, and the presence of a shadow may be misleading, as has been recently[2] shown, by the presence of calcareous deposits in the iliac vessels or lymphatic glands. Again, there is no uniform opinion as to the best method of reaching the calculus in the various positions in which it may be found.

Deaver[3] contributes an account of the subject with a report of five cases. He points out that calculus in the ureter is very much more common than was formerly supposed, indeed the Röntgen rays show it to be more common than in the kidney itself. In the descent from that organ it is prone to be arrested at three points: (1) Two inches from the pelvis of the kidney, as the ureter bends forward, over the psoas muscle; (2) at the brim of the pelvis where it dips down across the bifurcation of the common iliac artery; and (3) close to the

[2] *Brit. M. J.*, 1905, i. 1325. [3] *Ann. Surg.*, 1906, xlii. 732.

vesical orifice of the ureter. The symptoms of ureteral calculus are those of renal calculus, except when the stone is lodged in the lower end of the duct. The difficulty is to determine at what point the stone is lodged. Tenderness at the seat of impaction and a technically-perfect skiagram are valuable aids to diagnosis, but in cases of doubt the kidney should be explored first through the loin and a sound passed down the ureter. If after an attack of renal colic symptoms of renal calculus persist we may be certain at any rate that the stone has not travelled as far as the lower ureter. Although a calculus may remain lodged in the ureter indefinitely without producing serious symptoms, yet such cases are exceptional, and it is questionable whether it would not be the surgeon's duty to remove it as a prophylactic measure. If the stone is rough and mammillated it is more apt to excite ulceration and inflammation, though less apt to absolutely occlude the ureter than a smooth stone. On the other hand, a small stone is more likely to pass, but if it does become arrested is almost certain to give rise to hydronephrosis. The operation for removal of the calculi is satisfactory. One of the five cases reported died owing to stones being impacted in both ureters. The route to be chosen for removal is of much importance. If the stone is known to be near the bladder it is usually most successfully removed intravesically. In females it has been removed by the vagina, but this is less satisfactory, and in them the urethra can be dilated, the vesical orifice of the ureter incised, and the calculus removed with a suitable forceps. In males quite large stones have been removed by incising the lower end of the ureter through a suprapubic opening in the bladder. This means, however, will not suffice unless the calculus is lodged very close to the vesical orifice of the ureter. If impacted an inch away from the bladder wall, the extra-peritoneal route is the best. In this operation the ureter is carried along with the peritoneum as the latter is stripped up from the iliac fossa, and is to be sought on the vesical side of the wound. When a stone is exposed in the ureter, it is proper to try to dislodge it and push it either upward into the pelvis of the kidney or downwards into the bladder. An incision into the ureter itself is usually to be avoided, though in Deaver's opinion the probability of a permanent urinary fistula remaining is exaggerated. If the stone cannot be dislodged, it is safer to incise the ureter than to attempt to crush the stone *in situ*. The opening should be closed by two rows of sutures, catgut for the submucous tissues, and a silk suture of the Lembert type for the muscular wall, this plan diminishing the probability of a fistula persisting. Carcinoma was found immediately above the impaction in one of the cases recorded. At the conclusion of the paper the following unsettled points are put for discussion:—(1) The value of the X-ray and cystoscope with catheterising of the ureter in diagnosis; the significance of localised tenderness; the propriety of exploring the ureter intraperitoneally to locate the suspected stone. (2) The question of removal of quiescent or latent stones. (3) The best route for the removal of juxtavesical stones, whether perineal, vaginal, intravesical, suprapubic or extraperitoneal. (4) The proper treatment of the stone when found, whether it should be pushed on into the bladder, should be crushed, or whether the ureter should be incised. (5) The best method of suturing the ureter. (6) Whether nephrectomy is to be countenanced except for incurable disease.

Gibbon,[1] under the title, "The transperitoneal examination of the ureter in cases of suspected ureteral calculus and the combined intra- and extra-peritoneal uretero-lithotomy," illustrates certain advantages of a thorough palpation of the ureter when the abdomen is opened for lesion of other organs and specially for the less acute varieties of appendicitis. The first case quoted is that of a man, æt. 39, admitted as a case of subsiding appendicitis. He had had repeated attacks of pain in the abdomen, accompanied by vomiting and diarrhœa. There was persistent tenderness on deep pressure in the right iliac region, but no urinary symptoms. The urine showed a number of red-blood cells. The abdomen was opened through the right rectus, and whilst searching for the appendix a hard lump was felt below the iliac vessels in the true pelvis. The appendix was removed, there being evidence of past inflammation, and the wound being enlarged downwards, it was ascertained that the lump felt was a ureteral calculus. The peritoneum was now stripped downwards from the abdominal and pelvic wall to the ureter. The latter was opened longitudinally, and a good-sized stone removed. A gauze drain was passed down to the opening in the ureter and the peritoneal cavity tightly closed. The rest of the wound was closed except at its lower part. No suture was put in the ureter, but the fistula resulting was healed in a month. The second case was that of a woman, æt. 32, also admitted for appendicitis; history of repeated attacks of pain in the right iliac region, accompanied by vomiting; no urinary symptoms; urine contained a few red-blood cells and a trace of albumen. A similar operation was performed as in the previous case. The appendix, though normal, was removed, and a calculus then found in the ureter about the same position as before. It was dealt with in exactly the same way. Although a smaller opening had to be made in the ureter, there was a greater and more prolonged leakage of urine. The author points out the importance of palpating the ureter in all interval operations for appendicitis and cases of subacute or chronic inflammation of the uterine appendages. The immediate removal of a stone detected by palpation through the abdomen is not generally advocated, and when it has been done has been carried out through the peritoneum or extraperitoneally through a separate incision, the first one being closed. The author ventures to

[1] *Ann. Surg.*, 1906, xlii. 742.

think that his method has not previously been employed; he points out the great advantages to be derived from having a finger in the peritoneal cavity and on the stone during the exposure of the ureter in the extraperitoneal portion of the wound. There was no infection of the peritoneum in either of his cases, and he thinks with care it can be avoided. Another advantage from the combined intra- and extra-peritoneal method is that the stone can more easily be forced on into the bladder or back on to the dilated portion of the ureter. This method is not advocated for all cases, but in all doubtful cases, and when the abdomen has been opened for some other condition, the stone should be immediately removed either through a separate incision or after the manner just described. To remove it through the peritoneum involves too great a risk. In neither of the above cases was the ureter sutured, but the author thinks that in future cases he will close the incision and introduce a drain down to the sutures.

Heaton[1] reports the case of a little girl who first had a stone removed from the right kidney and was readmitted three years later for hæmaturia and pain in the left lumbar region. The left kidney was explored, but only granulation tissue found. A probe was passed a considerable distance down the ureter without meeting any obstruction. The symptoms persisted, and she was again admitted. An ill-defined fulness could be felt high up in the rectum to the left of the middle line. A radiograph located a stone low down in the pelvis. Nothing being felt on sounding the bladder, the ureter was exposed by a curved incision in the left iliac region as for ligature of the common iliac vessels. The peritoneum was pushed backwards and stripped up from the iliac fossa, carrying the ureter with it. The latter was much dilated and thickened. On opening it a stone was felt with a probe close to the entrance into the bladder. It was gently forced upwards and removed, the wound in the ureter closed, and the external wound drained. The patient made a good recovery. The author remarks that the calculus was probably present in the ureter at his second operation, but that he failed to pass the probe far enough to feel it. When impacted in this position in adult women it can usually be felt *per vaginam*, and may often be removed by this route. It is often a matter of difficulty to remove it from above when impacted so low down, owing to the ureter being surrounded at the base of the broad ligament by a plexus of large vessels and to the proximity to the uterine artery.

Campbell[2] points out the difficulties in the diagnosis of calculus in the urinary tract above the bladder, and mentions gallstones, appendicitis, intestinal obstruction in the early stages, true renal colic, and the crisis in floating kidney, as some of the conditions to be differentiated. He thinks the most important aid to diagnosis in conjunction with the pain is

[1] *Birmingh. M. Rev.*, 1906, lix. 137. [2] *Montreal M. J.*, 1906, xxxv. 266.

urinary analysis, and that the value of the X-rays has been exaggerated. He narrates the case of a woman of 33 who complained of pain in the lumbar region, radiating down both legs and into the abdomen. It began suddenly on the right side about the level of the umbilicus, causing vomiting and necessitating morphia. There was no subsequent jaundice, no blood in the urine, or retention. The patient remained well for a year, and then she began to have frequent similar attacks. She lost twenty-five pounds in two years. The attacks were never followed by any change in the urine. On examination in the right nipple line and slightly above the iliac crests a small not very tender area could be distinguished. A catheterised specimen of urine showed a trace of albumen, a small quantity of pus, and numerous crystals of calcium oxalate. Two skiagrams showed a definite shadow on the right side about an inch above the iliac crest. A lumbar incision was made extending forwards, and an impacted stone removed from a spot 1½ in. below the pelvis of the kidney. The wounds in the ureter and skin were closed, and the patient made an excellent recovery.

Hubbard[1] reports an interesting case of a man, æt. 48, admitted with pain in the right side of the abdomen, extending to the back. There was tenderness down the loin to McBurney's point. Whilst under observation he had a more severe attack of pain with vomiting. The diagnosis seemed to lie between a mild retro-cæcal appendicitis and a renal calculus, so the abdomen was opened through the right rectus. The appendix, though showing no sign of disease, was removed, and a small mass felt in the ureter. The abdominal incision was closed, the patient turned over, and the right kidney exposed through the loin and the ureter traced downwards. The stone was found about 1½ in. below the lower pole of the kidney. The ureter was incised, and the stone removed with some difficulty, owing to the firmness of the impaction. The incision in the ureter was closed with fine silk, and a gauze wick passed down to suture line and the wound closed. There was a little leakage of urine from the wound, and the resulting sinus was not entirely closed for nearly two months.

Bennett,[2] in referring to the use of X-rays in the diagnosis of abdominal pain, points out their great importance in cases where the symptoms point somewhat inconclusively to appendicitis, and relates two cases. The first, a girl of 18, admitted for attacks of appendicitis and tenderness over McBurney's spot, with abdominal resistance, in which the X-ray showed a good-sized calculus in the ureter; the other, a middle-aged man, who after eight attacks of appendicitis had his appendix removed. The operation had no effect on the attacks, but subsequently one of them terminated abruptly, and within three

[1] *Boston M. & S. J.*, 1905, cliii. 584.
[2] *Practitioner.* Extra X-ray number, 1906.

days a small stone was passed by the urethra and no further attacks followed. An X-ray examination might have shown this.

As bearing to some extent on Heaton's case, the writer had a small boy about a year ago admitted with symptoms of renal calculus. A skiagram showed very clearly two or three stones, and these were removed from the kidney, but the symptoms persisted after operation, and a few days later there was abdominal pain from distended bladder and a calculus impacted in the urethra. This was removed with relief to all the symptoms. On reference to the skiagram, a distinct shadow was seen on the brim of the pelvis corresponding in size with the stone removed from the urethra. This had escaped the observation of both skiagraphist and surgeon.

From a brief review of these recently-published cases one may deduce the following points: That the diagnosis of ureteral calculus is often fraught with difficulty, the symptoms on the one hand may be indistinguishable from those of renal calculus, and on the other hand there may be no symptoms whatever pointing to an affection of the urinary tract. That these latter are usually mistaken for intra-abdominal conditions of quite another nature, and most often for appendicitis. So likely is this to be the case that in interval operations for the latter condition it is recommended that the ureter be carefully palpated, and specially when obvious signs of disease of the appendix are absent. That when a calculus is discovered in the ureter it should be removed at once: if quite close to the bladder, through that viscus, or perhaps in a few cases in women by the vaginal route; otherwise by one of the many extra-peritoneal methods; that it should not be removed through the peritoneum; and, finally, that the ureter should be sutured when possible, but if impossible, it is unlikely that a permanent fistula will remain.

Harold F. Mole.

